# Quantitative flow ratio-guided staged percutaneous coronary intervention in patients with ST-segment elevation myocardial infarction

**DOI:** 10.1016/j.heliyon.2024.e39335

**Published:** 2024-10-13

**Authors:** Shenglong Hou, Xinxin Zhu, Qi Zhao, Huimin Xian, Kun Wang, Chao Qu, Ying Wang, Xin Jiang, Dongdong Qian, Yi Liu, Wei Zhou, Yuqing Wang, Lu Liu, Ruoxi Zhang, Qianfu Wu

**Affiliations:** a Department of Cardiology, Heilongjiang Provincial People's Hospital, 150036, Harbin, Heilongjiang, China; b Department of Cardiology, Second Hospital of Harbin Medical University, 150086, Harbin, Heilongjiang, China; c Department of Cardiology, First Hospital of Harbin Medical University, 150086, Harbin, Heilongjiang, China; d Department of Clinical Medicine, Jiecheng Medical, 215123, Su Zhou, Jiangsu, China; e Brise Pharmaceutical Co., LTD, Shanghai, 200071, China; f Department of Surgery, The affiliated Dazu's Hospital of Chongqing Medical University, 402360, Chongqing, Chongqing, China; g Department of Geriatrics, Shanghai Municipal Hospital of Traditional Chinese Medicine, Shanghai University of Traditional Chinese Medicine, Shanghai, 200071, China; h Department of Cardiology, Harbin Yinghua Hospital, 150100, Harbin, Heilongjiang, China; i Department of Intensive Care Unit, Longhua Hospital, Shanghai University of Traditional Chinese Medicine, Shanghai, China

**Keywords:** Quantitative flow ratio, Staged percutaneous coronary intervention, Non-infarct-related arteries, ST-Elevation myocardial infarction, Cost

## Abstract

**Background:**

The need for primary percutaneous coronary intervention (PCI) and staged PCI strategy for ST-segment elevation myocardial infarction (STEMI) with multivessel coronary disease is well documented. This study aimed to evaluate the efficiency, safety, and cost benefit of quantitative flow ratio (QFR)-guided staged PCI in patients with STEMI.

**Methods:**

We conducted a retrospective study involving 2256 patients meeting STEMI criteria having at least one lesion (≥50 %) in non-infarct-related (NIR) arteries. These patients had undergone primary PCI for infarct-related (IR) arteries and staged PCI for NIR arteries. Patients were categorized into two groups based on the strategy guided either by QFR or quantitative coronary angiography (QCA) as determined by the clinicians during primary PCI in real-world. For patients guided by QFR, a threshold of ≤0.80 serves as the cut-off value for determining the need for PCI. We recorded the demographics, clinical data, and QFR values of none-infarct-related arteries. The efficiency, safety, and cost benefit of the QFR-guided staged PCI were evaluated.

**Results:**

The QCA-guided group had a higher rate of Killip II. In the QFR-guided group, there was a higher proportion of left anterior descending coronary artery lesions in infarct-related arteries. The mean QFR value of non-infarct-related (NIR) arteries remained consistent at 0.83 across both groups, irrespective of whether the measurement was taken during the primary PCI or the staged PCI phase. Among patients with QFR ≤0.8, the QFR values during staged PCI were significantly higher than that during primary PCI, with a significantly greater increase compared to patients with QFR >0.8. The proportion of staged PCI, number of stents per patient, and cost of staged PCI per patient were significantly lower in the QFR-guided group compared to the QCA-guided group. In the long-term follow-up period, there were no statistically significant differences between the two groups in terms of major adverse cardiac events and clinic visits, except for target vessel revascularization.

**Conclusions:**

QFR resulted in a reduction in the proportion of STEMI patients with multivessel coronary disease undergoing invasive coronary angiography and staged PCI. Furthermore, it decreased the incidence of target vessel revascularization (TVR) and medical costs, without increasing major adverse cardiovascular events. Our future work will focus on large multi-center perspective studies for the feasibility of QFR guided staged PCI in patients with STEMI.

## Graphical abstract

**Figure:**
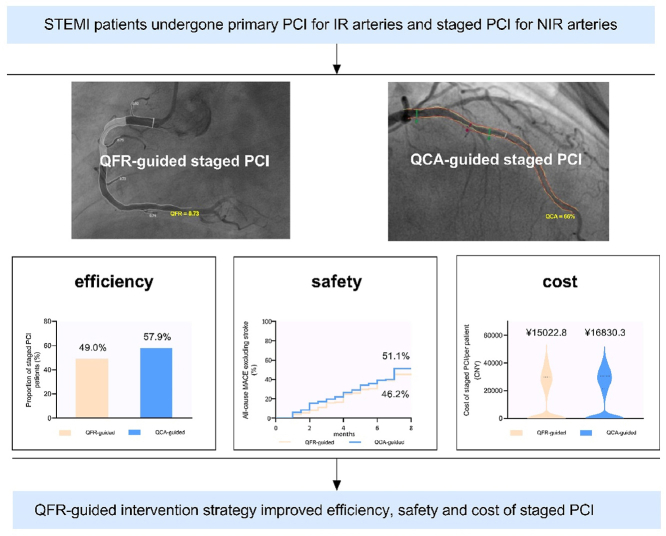

## 1 Introduction

Approximately 30–50 % of patients who need percutaneous coronary intervention (PCI) to treat ST-elevation myocardial infarction (STEMI) have extra stenotic coronary arteries in non-infarct-related (NIR) arteries that call for invasive treatment across stages of severity [1]. However, even when considering the investigation of NIR arteries in a staged procedure during the subacute STEMI phase, barriers such as repeated invasive procedures, associated risks, cost of pressure wires and vasodilators, insufficient financial reimbursement, and time constraints in the catheterization lab persist [2]. The most reliable approach to determining the need for PCI in NIR arteries is to functionally assess them during the primary PCI procedure.

There is accumulating evidence demonstrating the utility of fractional flow reserve (FFR) in evaluating the patients with coronary artery disease (CAD) [3]. Clinical trials have shown that an FFR value of 0.8 or less indicates a significant coronary lesion [4,5]. Additionally, revascularization guided by FFR has been shown to also reduce the cost of CAD management and the incidence of major adverse cardiac events (MACEs) compared to traditional approaches [6,7]. Although FFR improves clinical outcomes, the need for adenosine-induced hyperemia, extra catheterization with radiation exposure, and the cost of the pressure wire used for FFR measurement have limited the clinical adoption of this technique [8]. Alternative methods developed to calculate FFR include the measurement of quantitative flow ratio (QFR). QFR utilizes functional coronary imaging indices based on computational fluid dynamics (CFD) to potentially identify functionally significant lesions non-invasively, without requiring intracoronary instrumentation or adenosine administration. This approach avoids disruptions to the workflow of primary PCI for patients with STEMI. Growing evidence indicates good agreement between QFR and FFR in patients with stable CAD [9,10]. While recent years have seen an increasing number of studies investigating the potential role of QFR in functionally assessing NIR arteries in patients with STEMI, there remains a need for further research to fully establish its clinical utility [11]. Therefore, this study aimed to evaluate the efficiency, safety, and cost benefits of QFR-guided staged PCI in patients with STEMI.

## 2 Methods

### 2.1 Ethics statement

The research protocol was authorized by the local ethics committee of each institution and was carried out in accordance with the provisions of the Declaration of Helsinki (Approval number is SYDWGZR-2020-128). All patients provided written informed consent prior to participation in this study.

### 2.2 Study design and populations

This retrospective analysis included consecutive patients with STEMI who underwent primary or staged PCI at multiple centers, including Heilongjiang Provincial People's Hospital, Second Hospital of Harbin Medical University, First Hospital of Harbin Medical University, Harbin Yinghua Hospital, and Shanghai Municipal Hospital of Traditional Chinese Medicine. STEMI was diagnosed if a patient exhibited the following: chest pain for >30 min and 2 mm in at least two contiguous precordial electrocardiography (ECG) leads or >1 mm in at least two contiguous limb ECG leads or a newly developed left bundle branch block [12]. Inclusion criteria included patients with STEMI who reached to the PCI center within 90 min after onset of symptoms, for whom multivessel disease (at least 1 NIR lesion with ≥50 % lumen diameter stenosis) was diagnosed after coronary artery angiography, and who gave their written consent before inclusion [13]. The exclusion criteria included patients with STEMI with left main disease (stenosis >50 % by visual estimation), patients with history of coronary artery bypass graft (CABG) surgery, cardiogenic shock, patients with a life expectancy <2 years.

**Fig. 1:**
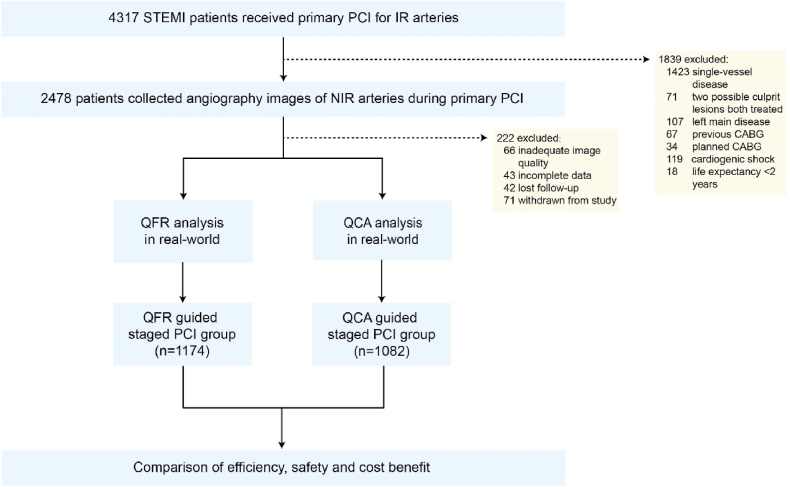
Study flowchart. PCI, percutaneous coronary intervention; NIR, non-infarction related; QFR, quantitative flow ratio; QCA, quantitative coronary angiography.

Between January 2016 and December 2018, 2256 patients with STEMI who underwent primary PCI based on QFR or QCA guidance were consecutively enrolled. These patients were divided into two groups depending on the strategy guided either by QFR or QCA as determined by the clinicians during primary PCI in real-world (Fig. 1). The inclusion criteria encompassed patients with STEMI who intended to undergo staged PCI in NIR arteries within a timeframe of 7–45 days following the index procedure. Standard clinical practice was followed for the treatment of all patients. Long-term follow-up, spanning at least 5 years, was conducted through retrospective electronic medical record review, outpatient records, and telephone follow-ups. The efficiency, safety and cost benefit of staged PCI were assessed in both groups. The present study was conducted in accordance with the Declaration of Helsinki. Ethics committees at each center approved the study, and all patients agreed to sign informed consent forms.

### 2.3 QCA and PCI

In the current study, all interventional data were evaluated by skilled interventional cardiologist [14]. According to previous study, NIR lesion was defined by vessel stenosis ≥50 %, where clinical decision-making was ≥70 % QCA stenosis [15]. The myocardial infarction thrombolysis (TIMI) test was performed in all patients only at the time of the first PCI. Baseline data for all patients were obtained by two people in a database at each center.

To ensure originality and avoid overlap with existing texts, the revised version of the provided content is as follows: Angiographic images were acquired using flat-panel angiographic systems, specifically the AXIOM Artis (Siemens Healthcare, Erlangen, Germany), Innova (GE, Wauwatosa, Wisconsin), AlluraXper (Philips, Amsterdam, Netherlands), and INTEGRIS Allura (Philips). These images were recorded at a rate of 15 frames per second. Prior to image acquisition, operators were given a table outlining the recommended angiographic views to ensure consistent imaging quality. Contrast medium was administered either manually with a steady, forceful injection or via a pump at an approximate flow rate of 4 ml/s. The methodology for the acquisition and analysis of these images has been previously detailed in the literature.

All patients were operated on collaboratively by two skilled interventional cardiologists following standard procedures. According to previous literature, all patients received standard antiplatelet therapy before interventional operation [16]. Post-operatively, all patients were transferred to our cardiac care unit and received standard treatment for STEMI, which consisted of 100 mg of aspirin, 20 mg of atorvastatin or 10 mg of rosuvastatin, and 75 mg of clopidogrel once a day or 90 mg of ticagrelor twice a day [17].

### 2.4 QFR evaluation

**Fig. 2:**
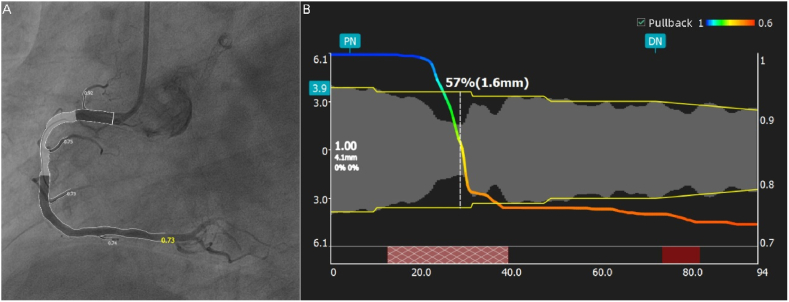
A representative case of non-infarct related artery, specifically the RCA (QFR = 0.73). (A) Two-dimensional images. (B) Functional assessment.

In this study, the most advanced AngioPlus 2.0 software (PULSE MEDICAL, Shanghai, China) was used to analyze coronary angiography. (Fig. 2). The Murray-law QFR (μQFR) software calculates the QCA value based on a single angiographic projection, incorporating the step-down reference diameter function, which is particularly advantageous in the analysis of bifurcation lesions. This method allows for a more precise assessment of lesion severity and decision-making during PCI. As such, we utilized the QCA values derived directly from the μQFR software during the QFR analysis to ensure consistency and accuracy in evaluating the coronary artery lesions [18].

According to the manufacturer's requirements, our software requires a capture of 15 frames per second to perform QFR. All QFR data is collected with the assistance of skilled engineers. After the doctor obtains a fixed QFR, they utilize the TIMI frame number to estimate the coronary flow of the contrast agent [18]. Based on TIMI data, the QFR value is automatically obtained [19]. For the clinical treatment decision-making, a cut-off value of ≤0.80 was used in this study, as it is recommended in description of the methods to use [13]. In the QFR-guidance group, the QFR values reported during the primary PCI were obtained on-site and recorded in the interventional procedure electronic report. And the decision to proceed with staged PCI was based on the QFR results obtained during the primary PCI. In both groups, the QFR analyses conducted during the primary PCI and staged PCI were performed independently, with the results from each phase being blinded to the other.

### 2.5 Simulation of stents guided by QFR and QCA

In this study, to simulate the difference in the number of stents guided by QFR and QCA, we employed the following methods.

(i) Data Collection and Processing

Initial coronary angiography images were used to analyze coronary artery lesions. For each patient, the quantitative flow ratio (QFR) values and coronary angiography (QCA) results were evaluated and recorded under both guidance methods.

(ii) Stent Number Simulation

QFR-Guided Group: Based on the results from the QFR analysis, the lesions requiring stent implantation were selected. For lesions with QFR ≤0.8, stent implantation was decided. The length and number of stents were calculated based on clinical experience and the anatomical structure of the coronary artery. QCA-Guided Group: Lesions requiring stent implantation were identified based on traditional coronary angiography visual assessment. The length and number of stents were determined according to the severity of coronary artery stenosis.

(iii) Blinded Analysis

To ensure the independence of the analysis, the decision-making process for stent implantation under QFR and QCA guidance was conducted independently and blinded. This means that the analyst in the QFR group did not have access to the results of the QCA group and vice versa.

(iv) Data Validation

All simulated stent numbers and positions were reviewed by two independent cardiology experts to ensure the reliability of the results.

### 2.6 Definitions

Reperfusion time was defined as the time from the onset of chest pain to balloon entry, and the door-to-balloon time was defined as the time from admission to balloon dilation. In this study, patients with diabetes, hypertension, and hyperlipidemia were independently diagnosed by two independent physicians according to the diagnostic criteria of today's latest guidelines. Smokers were defined as individuals who either continued to smoke or quit within 1 year. MACE included all-cause mortality, cardiovascular death, reinfarction, in-stent restenosis, stent thrombosis, target vessel revascularization, heart failure, rehospitalizations stroke, atrial fibrillation, ventricular arrhythmias, and atrioventricular block in hospital and follow-up.

### 2.7 Statistical analysis

For continuous data, the mean value is presented as a percentage, while categorical data are expressed as frequencies. Group comparisons were conducted using an independent two-sample *t*-test or Mann-Whitney *U* test for non-normal distribution data. Categorical variables were compared using either the Chi-square test or Fisher's exact test, as appropriate. To estimate survival status, the Kaplan-Meier (K-M) technique was utilized, and the log-rank test was used to compare survival distributions. To compare clinical outcomes between groups, we performed statistical adjustments using logistic regression and Cox hazards regression models. Statistical significance was set at two-sided p-values of <0.05. All statistical analyses were performed using SPSS version 22.0 (SPSS Inc., Chicago, IL, USA).

## 3 Results

### 3.1 Baseline characteristics

The screening process involved 4317 patients, among whom 2256 were finally enrolled in the study between January 2016 and December 2018. A comparison of the two groups indicated a difference regarding Killip class, as patients had a higher rate of Killip II in the QCA-guided group (*P* = 0.005). There was no significant difference in other characteristics of the two group's baseline demographics (Table 1).

**Table 1:** Basic characteristics in patients.

	QFR-guided (n = 1174)	QCA-guided (n = 1082)	*P*-value
Age (years)	64.0 (6.2)	63.9 (6.0)	0.852
Male sex	751 (64.0)	714 (66.0)	0.331
BMI (kg/m^2^)	26.1 (3.0)	25.9 (3.0)	0.126
Cardiovascular risk factors			
Hypertension	752 (64.1)	695 (64.2)	0.965
Diabetes mellitus	221 (18.8)	202 (18.7)	0.957
Hyperlipidemia	699 (59.5)	676 (62.3)	0.154
Current smoking	421 (35.9)	361 (33.4)	0.215
Previous MI	124 (10.6)	115 (10.6)	1.000
Previous PCI	113 (9.6)	106 (9.8)	0.943
Killip class on admission			
I	879 (74.9)	796 (73.6)	0.500
II	161 (13.7)	195 (18.0)	0.005
III	95 (8.1)	67 (6.2)	0.087
IV	39 (3.3)	24 (2.2)	0.125
Laboratory data			
SBP (mmHg)	119.9 (19.4)	120.2 (19.3)	0.693
DBP (mmHg)	73.0 (14.0)	72.8 (13.6)	0.749
Peak Troponin I (μg/L)	121.4 (16.4)	118.6 (13.8)	0.646
Peak CK-MB (μg/L)	30.46 (13.50)	30.53 (13.54)	0.904
NT-proBNP (pg/mL)	702.2 (71.0)	700.2 (75.4)	0.520
Total cholesterol (mol/L)	4.50 (0.77)	4.50 (0.80)	0.788
LDL-cholesterol (mol/L)	3.08 (1.10)	3.01 (1.11)	0.156
HDL-cholesterol (mol/L)	1.29 (0.49)	1.30 (0.50)	0.475
Triglyceride (mol/L)	2.15 (1.01)	2.19 (1.01)	0.427
LVEF (%)	52.3 (9.4)	53.5 (8.7)	0.320
Medications at discharge			
Aspirin	1099 (93.6)	1017 (94.0)	0.727
Clopidogrel	740 (63.0)	714 (66.0)	0.146
Ticagrelor	434 (37.0)	368 (34.0)	0.146
Statin	1166 (99.3)	1080 (99.8)	0.112
β-Blocker	728 (62.0)	650 (60.1)	0.364
ACEI/ARB	469 (39.9)	433 (40.1)	1.000
Proton pump inhibitor	325 (27.7)	310 (28.7)	0.639

Data are presented as mean (SD) or n (%). QFR, quantitative flow ratio; QCA, quantitative coronary angiography; BMI, body mass index; STEMI, ST-elevation myocardial infarction; PCI, percutaneous coronary intervention; SBP, systolic blood pressure; DBP, diastolic blood pressure; CK-MB, creatine kinase-MB; HDL, high-density lipoprotein; LDL, low-density lipoprotein; NT-proBNP, N-terminal pro-brain natriuretic peptide; LVEF, left ventricular ejection fraction; ACEI angiotensin-converting enzyme inhibitor; ARB angiotensin receptor II blocker.

### 3.2 Coronary angiography, QFR, and PCI

For infarct-related arteries, a higher proportion of left anterior descending coronary artery lesion was noted in the QFR-guided group (*P* = 0.029) (Table 2). The mean QFR value of NIR arteries was 0.83 for both the primary and staged PCI (Table 3). There was no significant difference in the characteristics of the two group's NIR arteries.

**Table 2:** Coronary angiography and primary interventional therapy.

	QFR-guided (n = 1174)	QCA-guided (n = 1082)	*P*-value
Reperfusion time (min)	283.0 (118.8)	283.3 (115.9)	0.956
Door-to-balloon time (min)	67.4 (13.7)	67.1 (14.1)	0.664
Culprit lesion localization			
LAD	511 (43.5)	421 (38.9)	0.029
LCX	242 (20.6)	241 (22.3)	0.355
RCA	329 (28.0)	332 (30.7)	0.179
Diagonal branch	59 (5.0)	53 (4.9)	0.923
Obtuse branch	33 (2.8)	35 (3.2)	0.622
Stents per patient	0.95 (0.28)	0.95 (0.27)	0.783
Femoral access	117 (9.97)	112 (10.35)	0.780
IABP	117 (9.97)	112 (10.35)	0.780

Data are presented as mean (SD) or n (%). QFR, quantitative flow ratio; QCA, quantitative coronary angiography; LAD, left anterior descending coronary artery; LCX, left circumflex coronary artery; RCA, right coronary artery; IABP, intra-aortic balloon pump.

**Table 3:** Non-infarct related arteries characteristics.

	QFR-guided (n = 1174)	QCA-guided (n = 1082)	*P*-value
NIR lesion localization			
LAD	399 (34.0)	375 (34.7)	0.756
LCX	577 (49.1)	514 (47.5)	0.448
RCA	528 (45.0)	499 (46.1)	0.612
Diagonal branch	189 (16.1)	202 (18.7)	0.119
Obtuse branch	113 (9.6)	98 (9.1)	0.665
Bifurcation	300 (25.6)	295 (27.3)	0.364
Heavy calcification	258 (22.0)	206 (19.0)	0.086
Lesion geometry			
MLD (mm)	1.18 (0.23)	1.17 (0.23)	0.337
DS (%)	65.1 (8.8)	64.9 (9.2)	0.726
Lesion length (mm)	18.3 (7.5)	18.3 (7.7)	0.891
QFR value for primary PCI	0.83 (0.05)	0.83 (0.07)^a^	0.471
QFR value for staged PCI	0.83 (0.05)^a^	0.83 (0.07)^a^	0.257

Data are presented as mean (SD) or n (%). NIR, non-infarct related; LAD, left anterior descending coronary; LCX, left circumflex coronary; RCA, right coronary; QFR, quantitative flow ratio; QCA, quantitative coronary angiography; PCI, percutaneous coronary intervention, DS, diameter stenosis; MLD, minimal luminal diameter.

a QFR value did not influence the clinician's decision-making process, which were calculated retrospectively based on the angiographic results.

**Fig. 3:**
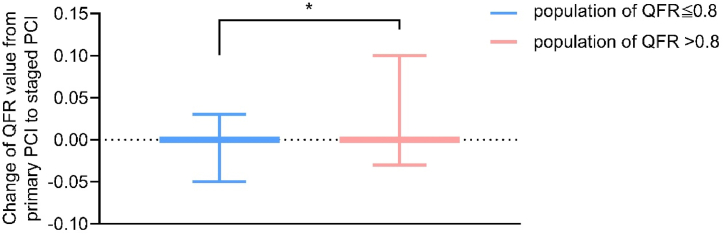
Comparison of changes in QFR between the group with QFR >0.8 and the group with QFR ≤0.8 during primary PCI and staged PCI. QFR, quantitative flow ratio; PCI, percutaneous coronary intervention. ∗*P* < 0.001, non-normal distribution data was assessed using Mann-Whitney *U* test.

The comparison of QFR between primary and staged PCI groups is shown in Table 4. In the population with QFR ≤0.8, the QFR values during staged PCI were significantly higher than those during primary PCI (0.77 vs. 0.76, *P* = 0.008). The percentage of cases where the strategic plan was significantly impacted due to differences in QFR values between primary and staged PCI (58.0 % vs. 53.2 %, *P* = 0.001). Patients with a QFR ≤0.8 exhibited a significantly greater increase in QFR values during the staged PCI phase compared to patients with a QFR >0.8. Patients with QFR ≤0.8 demonstrated a significantly greater increase in QFR compared to patients with QFR >0.8 (*P* < 0.001, Fig. 3). Multivariate logistic regression analysis showed that QFR guided (odds ratio [OR] = 0.698, 95 % confidence interval [CI]: 0.591–0.824, *P* = 0.001] was independently associated with staged PCI plan (Table S1).

**Table 4:** Comparison of QFR between primary and staged PCI groups.

	Primary PCI (n = 2256)	Staged PCI (n = 2256)	*P*-value
QFR >0.8	0.86 (0.04)	0.86 (0.04)	0.795
QFR ≤0.8	0.76 (0.03)	0.77 (0.03)	0.008
Proportion of patients with QFR ≤0.8	1105 (49.0)	1024 (45.4)	0.158
Staged PCI strategy plan	1309 (58.0)	1201 (53.2)	0.001

Data are presented as mean (SD) or n (%). QFR, quantitative flow ratio; PCI, percutaneous coronary intervention.

**Fig. 4:**
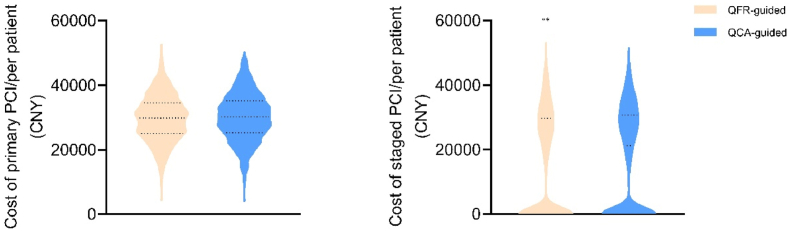
Comparison of cost on primary and staged PCI. ∗∗*P* = 0.018, non-normal distribution data was assessed using Mann–Whitney *U* test. PCI, percutaneous coronary intervention.

**Fig. 5:**
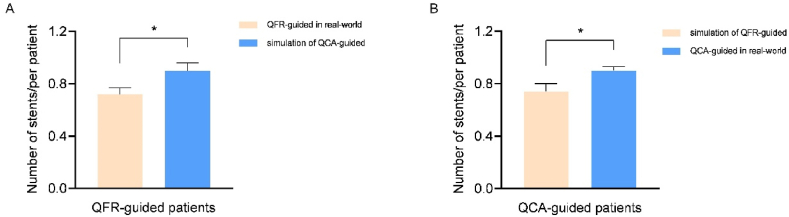
Comparison of staged PCI proportions and number of stents per patient. (A) Comparison of number of stents per patient in QFR-guided patients. (B) Comparison of number of stents per patient in QCA-guided patients. PCI, percutaneous coronary intervention.

Proportion of staged PCI, number of stents per patient, and cost of staged PCI per patient were significantly lower in the QFR-guided group than in the QCA-guided group (49.0 % vs. 57.9 %, *P* < 0.001, 0.72 vs. 0.84, *P* < 0.001 and 15022.8 CNY vs. 16830.3 CNY, *P* = 0.018; Table 5 and Fig. 4). In the QFR-guided group, the number of stents actually implanted was significantly lower than the simulated number of stents under QCA guidance (*P* < 0.001, Fig. 5A). Conversely, in the QCA-guided group, the number of stents actually implanted was significantly higher than the simulated number of stents under QFR guidance (*P* < 0.001, Fig. 5B).

**Table 5:** Comparison of staged PCI proportions and number of stents per patient.

	QFR-guided (n = 1174)	QCA-guided (n = 1082)	*P*-value
Proportion of staged PCI patients, n (%)			
Staged PCI	575 (48.98)	626 (57.86)	<0.001
Non-staged PCI	599 (51.02)	456 (42.14)	<0.001
Number of stents/per patient	0.72 (0.02)	0.84 (0.03)	<0.001

Data are presented as mean (SD) or n (%). PCI, percutaneous coronary intervention.

### 3.3 Clinical outcomes in long-term follow-up

**Fig. 6:**
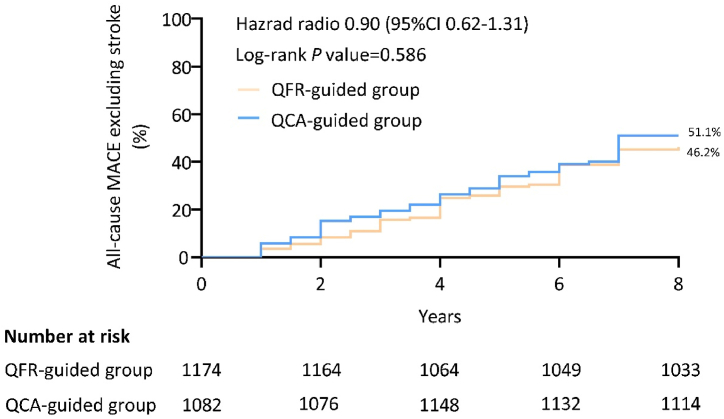
Kaplan-Meier curves for all-cause MACE excluding stroke. MACE, major adverse cardiac event.

The characteristics of the two group's clinic visits and MACE in long-term follow-up are shown in Table 6. The TVR rate in the QFR-guided group was significantly lower than that in the QCA-guided group (*P* = 0.036). The K-M curves for all-cause MACE excluding stroke is shown in Fig. 6. No significant difference was observed between the two groups (*P* = 0.586). Using the Cox regression model to analyze the adjusted variables (QFR guided, Age, Sex, Hypertension, Diabetes mellitus, Hyperlipidemia, Current smoking) on survival time, the results showed that none of these variables reached statistical significance (Table S2).

**Table 6:** Clinical outcomes in long term follow-up.

	QFR-guided (n = 1174)			QCA-guided (n = 1082)			*P*-value^a^
	A: Total	A1: IR	A2: NIR	B: Total	B1: IR	B2: NIR	
Clinic visits (times)	5.94 (0.99)	6.53 (0.88)	5.33 (1.04)^a^	5.93 (0.99)	6.80 (0.74)	5.12 (0.94)^b^	0.849
MACE (%)							
All-cause mortality^b^	72 (6.13)	21 (1.79)	11 (0.94)	57 (5.27)	18 (1.66)	12 (1.11)	0.414
Cardiovascular death^b^	52 (4.42)	21 (1.79)	11 (0.94)	44 (4.07)	18 (1.66)	12 (1.11)	1.000
Re-infarction	69 (5.88)	53 (4.51)	16 (1.36)^a^	54 (4.99)	44 (4.07)	10 (0.92)^b^	0.404
ISR	81 (6.90)	43 (3.66)	38 (3.24)	98 (9.06)	52 (4.81)	46 (4.25)	0.061
Stent thrombosis	8 (0.68)	8 (0.68)	0 (0.00)^a^	16 (1.48)	14 (1.29)	2 (0.18)^b^	0.098
TVR	67 (5.71)	37 (3.17)	30 (2.56)	86 (7.95)	49 (4.53)	37 (3.42)	0.036
HF	120 (10.22)	72 (6.13)	58 (4.94)	106 (9.80)	64 (5.91)	42 (3.88)^b^	0.779
Stroke	137 (11.67)	–	–	143 (13.22)	–	–	0.278
Re-hospitalization	323 (27.51)	211 (17.97)	112 (9.54)^a^	291 (26.89)	198 (18.30)	93 (8.60)^b^	0.776
New onset AF	130 (11.07)	–	–	130 (12.01)	–	–	0.510
VA	108 (9.20)	–	–	121 (11.18)	–	–	0.125
AVB	27 (2.30)	–	–	21 (1.94)	–	–	0.564

Data are presented as mean (SD) or n (%). NIR, NIR, non-infarction related; MACE, major adverse cardiovascular event; ISR, in-stent restenosis; TVR, target vessel revascularization; HF, heart failure; AF, atrial fibrillation; VA, ventricular arrhythmias; AVB, advanced atrioventricular block.

a The P-value is the result between A and B groups.^a^*P* < 0.05 between A1 and A2 groups;^b^*P* < 0.05 between B1 and B2 groups.

b The data for the IR and NIR groups were obtained from patients who underwent coronary angiography or had clear evidence from diagnostic tests, such as electrocardiograms and Holter monitoring, confirming the location of the culprit or non-culprit lesions responsible for fatal events.

In the QFR-guided group, cardiovascular death, re-infarction, stent thrombosis, and re-hospitalization rates were elevated in the infarction-related (IR) subgroup. Similarly, in the QCA-guided group, re-infarction, stent thrombosis, re-hospitalization, and heart failure (HF) rates were significantly higher in the IR subgroup compared to the NIR subgroup (Table 6).

## 4 Discussion

In this study, we aimed to evaluate the efficiency, safety, and cost benefits of QFR-guided staged PCI in patients with STEMI and multivessel disease. The key findings are as follows: 1) Compared to the QCA-guided group, the QFR-guided group had a lower proportion of staged PCI procedures, fewer stents per patient, and lower medical costs per patient for staged PCI. 2) During long-term follow-up, there were no significant differences between the two groups in major adverse cardiovascular events and clinic visits, except for a lower rate of target vessel revascularization in the QFR-guided group.

An increasing number of studies are currently focusing on treatment strategies for NIRs lesions in patients with STEMI with multi-vessel lesions. These studies have shown that preventive PCI in NIR arteries can reduce subsequent adverse events. Accumulating evidence indicating that strategies relying solely on angiographic evaluation to guide both primary and staged PCI have substantial limitations. Even now, advanced endovascular imaging methods, such as OCT, considerably aid interventional cardiologist in selecting their treatment strategies. However, inconsistencies between anatomical structure and coronary physiological function have been observed in many patients. This highlights the value of coronary physiology in PCI [20]. In recent years, FFR measurement has been able to evaluate the residual functional ischemic load, which was not possible with traditional endovascular imaging. Furthermore, several studies have also confirmed the unique value of FFR in predicting the poor clinical prognosis of patients with ACS [21,22]. However, FFR remains underutilized in clinical practice due to equipment and expensive guide wire, physician preferences, and risk of related complications. At present, QFR techniques using imaging are available to calculate FFR values, significantly mitigating these drawbacks [23,24]. Despite numerous clinical trials, uncertainty persists regarding the comparison between physiology-guided PCI and angiography-guided PCI alone in the context of acute myocardial infarction with multivessel disease. In fact, a recent multicenter randomized clinical trial investigating the superiority of FFR-guided PCI failed to demonstrate added benefits of physiology-guided PCI over angiography alone-guided PCI ^24^. This may be the primary reason for the non-superiority of FFR-guided PCI in patients with STEMI in the FLOWER-MI clinical trial, which also raised concerns regarding the reliability of vascular physiological assessment during the acute phase. The reliability of QFR in stable patients is well-established and has been repeatedly demonstrated in various studies that compared it to both hyperemic and resting indices [25,26]. It has been shown that acute QFR compared to staged QFR has higher diagnostic performance than acute FFR versus staged FFR or acute instantaneous wave-free ratio (iFR) versus staged iFR [27]. Additionally, studies have demonstrated excellent numerical agreement between QFR measured during primary PCI for STEMI culprit lesions and in cases of QFR measured in a staged procedure at least 3 months later [2,13]. This indicates agreement in both numerical values and clinical decision-making between measurements acquired during the acute phase of myocardial infarction and later during the stable phase. In our study, the initial experimental design was based on real-world conditions, utilizing the QFR values measured from NIR arteries during primary PCI as the basis for determining whether to proceed with staged PCI. It is undeniable that factors such as elevated left ventricular end-diastolic pressure (LVEDP) [27,28], decreased inflammation levels [29], and condition stabilization may influence endothelial function, thereby affecting the QFR values at the time of staged PCI. Consequently, in the latter phase of the trial, we further sought patient consent to acquire coronary angiography data during the staged PCI phase and calculated the corresponding QFR values. Comparing the QFR data between the primary PCI and staged PCI phases revealed no significant difference in overall QFR values, and the proportion of patients with QFR ≤0.8 did not show a notable change. This suggests that using QFR values from the primary PCI as the basis for determining whether to proceed with staged PCI, as opposed to using data from the staged PCI, did not alter the clinical decision-making process regarding performing PCI. Moreover, the QFR increment is significantly higher in the group with QFR ≤0.8 compared to the group with QFR >0.8. This suggests that lesions with poorer endothelial function demonstrate more significant improvement as acute myocardial infarction stabilizes. In the current literature, we have not identified other studies that address this phenomenon, nor have we obtained a complete mechanism to explain it, thus warranting further investigation. To our knowledge, this is the first study that comprehensively analyze the efficiency, safety, and cost-effectiveness of staging PCI in QFR-guided STEMI patients. In the present study, QFR guide reduced the proportion of staged PCI and number of stents per patient. This implies that QFR assessment could prevent unnecessary staged PCI in NIR arteries that coronary angiography considered significant but were not physiologically significant. Similarly, Kiyohara et al. performed a network meta-analysis comparing FFR, iFR, QFR, and angiography for guiding deferral of PCI. The key findings were that QFR was associated with slightly less reduction in major adverse cardiovascular events compared to FFR [30]. QFR-guided management was associated with significantly lower costs over 1 year of follow-up. The absolute difference in mean per-patient costs at 1 year in QFR-guided patients was numerically greater than in patients with QCA guide (15022.8 CNY vs. 16830.3 CNY), consistent with the low use of invasive angiography and staged PCI. High medical costs are a major barrier to medical care for many patients, especially in economically underdeveloped areas of developing countries. By lowering medical expenses, healthcare systems can alleviate the financial hardship associated with treatment [31]. Ensuring affordability of care for patients is essential for establishing a sustainable and ethical healthcare system [32].

In our study, although we could not directly access and analyze OCT or IVUS data, it is worth considering the potential implications of OCT-based FFR (OFR) and IVUS-based FFR (UFR) in the evaluation of culprit lesions during primary PCI [33]. OCT and IVUS provide detailed imaging of coronary artery morphology, which can complement functional assessments like FFR. These imaging modalities might offer additional insights when used alongside QFR, particularly in identifying the functional significance of lesions [34]. However, further studies are necessary to establish the clinical efficacy and utility of OFR and UFR in guiding PCI decisions in patients with STEMI.

### 4.1 Limitations

Our study has several limitations. First, there is a potential for significant referral bias due to the retrospective nature of the study, wherein both physicians and patients were not blinded to the QFR results and whether revascularization was performed. This inherent limitation may affect the generalizability and robustness of our findings, highlighting the need for cautious interpretation of the results. Second, our study is an observational study without statistical adjustments, which restricts the robustness of the data regarding the efficacy and safety of QFR guidance compared with QCA guidance. Third, some patients were excluded due to the patient's culprit lesion. This limitation renders our findings not universally applicable to all patients with STEMI. Fourth, nitrates are regularly administered before coronary angiography, but this could not be done in the acute phase of STEMI due to potential hemodynamic instability. This would lead to potential underestimation of coronary diameters and consequently lower values of QFR to an extent. Finally, QFR analysis was performed on the index coronary angiogram obtained during the initial emergency catheterization for STEMI, rather than waiting until the staged procedure. Due to factors such as elevated LVEDP, decreased inflammation levels, and the stabilization of the condition, the QFR value at this point may change compared to the acute phase.

## 5 Conclusions

QFR resulted in a reduction in the proportion of STEMI patients with multivessel coronary disease undergoing invasive coronary angiography and staged PCI. Furthermore, it decreased the incidence of TVR and medical costs, without increasing major adverse cardiovascular events. Our future work will focus on large multi-center perspective studies for the feasibility of QFR guided staged PCI in patients with STEMI.

## CRediT authorship contribution statement

**Shenglong Hou:** Data curation. **Xinxin Zhu:** Investigation. **Qi Zhao:** Data curation. **Huimin Xian:** Methodology. **Kun Wang:** Investigation. **Chao Qu:** Data curation. **Ying Wang:** Investigation. **Xin Jiang:** Data curation. **Dongdong Qian:** Data curation. **Yi Liu:** Investigation. **Wei Zhou:** Data curation. **Yuqing Wang:** Methodology. **Lu Liu:** Data curation. **Ruoxi Zhang:** Writing – review & editing, Writing – original draft, Funding acquisition, Conceptualization. **Qianfu Wu:** Validation, Supervision.

## Availability of data and materials

The raw data supporting the conclusions of this article will be made available by the authors, without undue reservation.

## Disclosures

The authors have no conflicts of interest to disclose.

## Funding sources

Support for this research comes from the 10.13039/501100001809National Natural Science Foundation of China (No. 81970297 and 82270319).

## Declaration of competing interest

The authors have no conflicts of interest to disclose.
