# Supplementary material for: Quantitative flow ratio-guided staged percutaneous coronary intervention in patients with ST-segment elevation myocardial infarction
Source: Heliyon. 2024 Oct 13;10(20):e39335. doi: 10.1016/j.heliyon.2024.e39335 (PMC11620227; doi:10.1016/j.heliyon.2024.e39335)
Supplement: Multimedia component 1 [file mmc1.docx]

**Table S1. Univariate and multivariate logistics regression analysis for staged PCI plan**

|  | Univariate | | Multivariate | |
| --- | --- | --- | --- | --- |
|  | OR (95% CI) | *P*-value | OR (95% CI) | *P*-value |
| QFR guided | 0.699 (0.592-0.826) | 0.001 | 0.698 (0.591-0.824) | 0.001 |
| Age | 0.993 (0.979-1.006) | 0.294 | 0.993 (0.980-1.007) | 0.337 |
| Sex | 0.993 (0.835-1.181) | 0.936 | 0.994 (0.835-1.184) | 0.949 |
| Hypertension | 1.029 (0.866-1.222) | 0.746 | 1.003 (0.841-1.195) | 0.997 |
| Diabetes mellitus | 1.083 (0.876-1.339) | 0.461 | 1.076 (0.869-1.333) | 0.503 |
| Hyperlipidemia | 1.110 (0.937-1.315) | 0.226 | 1.088 (0.917-1.292) | 0.335 |
| Current smoking | 1.168 (0.981-1.390) | 0.081 | 1.169 (0.981-1.394) | 0.082 |

PCI, percutaneous coronary intervention; OR, Odds Ratio; CI, Confidence Interval; QFR, quantitative flow ratio.
