# Supplementary material for: Quantitative flow ratio-guided staged percutaneous coronary intervention in patients with ST-segment elevation myocardial infarction
Source: Heliyon. 2024 Oct 13;10(20):e39335. doi: 10.1016/j.heliyon.2024.e39335 (PMC11620227; doi:10.1016/j.heliyon.2024.e39335)
Supplement: Multimedia component 2 [file mmc2.docx]

**Table S2. Univariate and multivariate COX regression analysis for all-cause MACE excluding stroke**

|  | Univariate | | Multivariate | |
| --- | --- | --- | --- | --- |
|  | HR (95% CI) | *P*-value | HR (95% CI) | *P*-value |
| QFR guided | 1.169 (0.826-1.654) | 0.379 | 1.170 (0.826-1.657) | 0.377 |
| Age | 0.987 (0.960-1.016) | 0.378 | 0.988 (0.960-1.016) | 0.388 |
| Sex | 0.911 (0.638-1.302) | 0.609 | 0.925 (0.647-1.324) | 0.671 |
| Hypertension | 1.118 (0.775-1.612) | 0.551 | 1.090 (0.753-1.579) | 0.647 |
| Diabetes mellitus | 1.262 (0.835-1.908) | 0.270 | 1.242 (0.819-1.881) | 0.307 |
| Hyperlipidemia | 1.159(0.808-1.661) | 0.423 | 1.144 (0.796-1.645) | 0.466 |
| Current smoking | 1.050 (0.733-1.506) | 0.790 | 1.017 (0.707-1.461) | 0.929 |

MACE, major adverse cardiac events; HR, Hazard Ratio; CI, Confidence Interval; QFR, quantitative flow ratio.
